# Occlusal changes during orthognathic therapy using a completely customised lingual appliance - initial retrospective observations

**DOI:** 10.1007/s00784-025-06347-9

**Published:** 2025-04-25

**Authors:** Stephan Christian Möhlhenrich, Jannik Steur, Sachin Chhatwani, Frauke Beyling, Gholamreza Danesh, Dirk Wiechmann

**Affiliations:** 1 Department of Orthodontics, Witten/Herdecke University, Alfred-Herrhausen-Str. 45, 58455 Witten, Germany; 2 Private Practice, Lindenstraße 44, 49152 Bad Essen, Germany; 3 Department of Orthodontics, Hannover Medical School (MHH), Carl-Neuberg-Straße 1, 30625 Hannover, Germany

**Keywords:** CCLA, Completely customised lingual appliances, Lingual orthodontics, Orthognathic surgery, PAR index, PAR score, Tooth contact, Treatment outcome

## Abstract

**Objective:**

The aim of this study was to examine the treatment outcomes, particularly the orthodontic decompensation before surgery, in patients who underwent orthognathic treatment and were treated with completely customised lingual appliances (CCLAs).

**Methods:**

25 patients who received combined orthognathic treatment for skeletal Class II (*N* = 10) or Class III (*N* = 15) malocclusion were retrospectively investigated. Study models from before treatment (T0), immediately before surgery (T1) and after treatment (T2), as well as digitised setup and operation models, were analysed using PAR Index and by measuring tooth pairs in contact.

**Results:**

The initial PAR scores were comparable (T0: Class II: 33.30 ± 7.85; Class III: 35.90 ± 1.20; *p* = 0.539), and significant improvements were observed following treatment (T0 vs. T2: Class II: *p* = 0.002; Class III: *p* = 0.002). Excellent final PAR scores were recorded, with no statistical differences between the groups (T2: Class II: 1.60 ± 2.80; Class III: 0.80 ± 2.08; *p* = 0.246). The PAR scores for setup and operation models were 0.00 ± 0.00 and 4.20 ± 3.29 for Class II and 0.07 ± 0.26 and 2.47 ± 1.92 for Class III, respectively. No significant differences were found between setup and final model (Class II: *p* = 0.063; Class III: *p* = 0.125), but between OP and final model (Class II: *p* = 0.002; Class III: *p* = 0.001). Tooth pairs in contact demonstrated similar results.

**Discussion:**

Using CCLAs in combined orthognathic treatment is efficient and leads to high-quality outcomes in Class II and III patients. Moreover, it enables excellent dental arch alignment before surgery.

**Clinical relevance:**

Optimal pre-surgical dental arch alignment, both before orthognathic surgery and after the completed therapy, can be achieved using CCLAs.

## Introduction

Patients scheduled to undergo orthognathic surgery generally receive pre-surgical orthodontic treatment [1]. Pre-surgical orthodontic decompensation involves intentionally reversing dentoalveolar compensations to correctly align the teeth with the anticipated post-surgical jaw configuration. In cases of skeletal Class II malocclusion, primarily for the division II/2, orthodontic decompensation necessitates increasing the overbite. In addition, in the presence of a dentoalveolar deep bite, the elongation of the front teeth must also be considered during decompensation if increasing the lower face height postoperatively would be meaningful [2]. Conversely, for skeletal Class III malocclusion, decompensation involves reducing the overbite below the maximum. Insufficient orthodontic decompensation prior to surgery significantly restricts the extent of surgical correction and compromises both aesthetic and functional outcomes, as dental interferences may occur that prevent the ideal positioning of the skeletal subunits [3]. The pre-surgical occlusion determines and influences the surgical bony segment displacements and can even limit them, which can result in unstable outcomes, extended postoperative treatment periods and dissatisfaction among patients and medical practitioners [4]. In this context, it remains unclear whether pre-surgical orthodontic treatment is more effective in Class II or Class III patients [5, 6]. Quast et al. reported that incisor decompensation is regardless of the underlying malocclusion often insufficient in all three dimensions and that surgical movements affect incisor inclination; therefore, they concluded that incisor inclination should be considered during pre-surgical orthodontic decompensation [5].

Effective torque control of the anterior teeth and posterior transverse correction, even in more severe cases, can be clinically achieved using completely customized lingual appliances (CCLAs) [7–13]. In general orthodontics, this technique is known for its high accuracy in achieving a final occlusion that closely matches the planned occlusion in the setup model [14–18]. The predictability of the post-treatment results achieved with CCLAs could also be advantageous in the combined orthodontic and surgical management of severe malocclusions. Specifically, the precise control of tooth positioning provided by the use of CCLAs may facilitate achieving ideal pre-surgical decompensation, a stable occlusion immediately after surgery and the retention of skeletal movements. Several case reports have highlighted the compatibility of orthodontic treatment and orthognathic surgery using lingual braces [19–31]. However, systematic research on this topic is not exiting.

Therefore, the primary aim of this retrospective study was to evaluate the overall treatment, while the secondary objective was to examine and compare the orthodontic alignment before surgical intervention considering the optimal dental arch form. Furthermore, it remains unclear whether pre-surgical orthodontic treatment is more effective in Class II or Class III patients [5, 6], and as consequence, certainly not the effect of CCLAs in this context. Thus, the significance of the underlying skeletal malocclusion was investigated as well as the degree to which CCLAs could achieve the planned setup in the context of combined orthodontic–orthognathic therapy assessed.

## Materials and methods

This study was conducted in accordance with the tenets of the Declaration of Helsinki. The Ethics Committee of the Medical Faculty of Witten/Herdecke University, Germany, reviewed and approved the study protocol (S-210/2024).

**Fig. 1:**
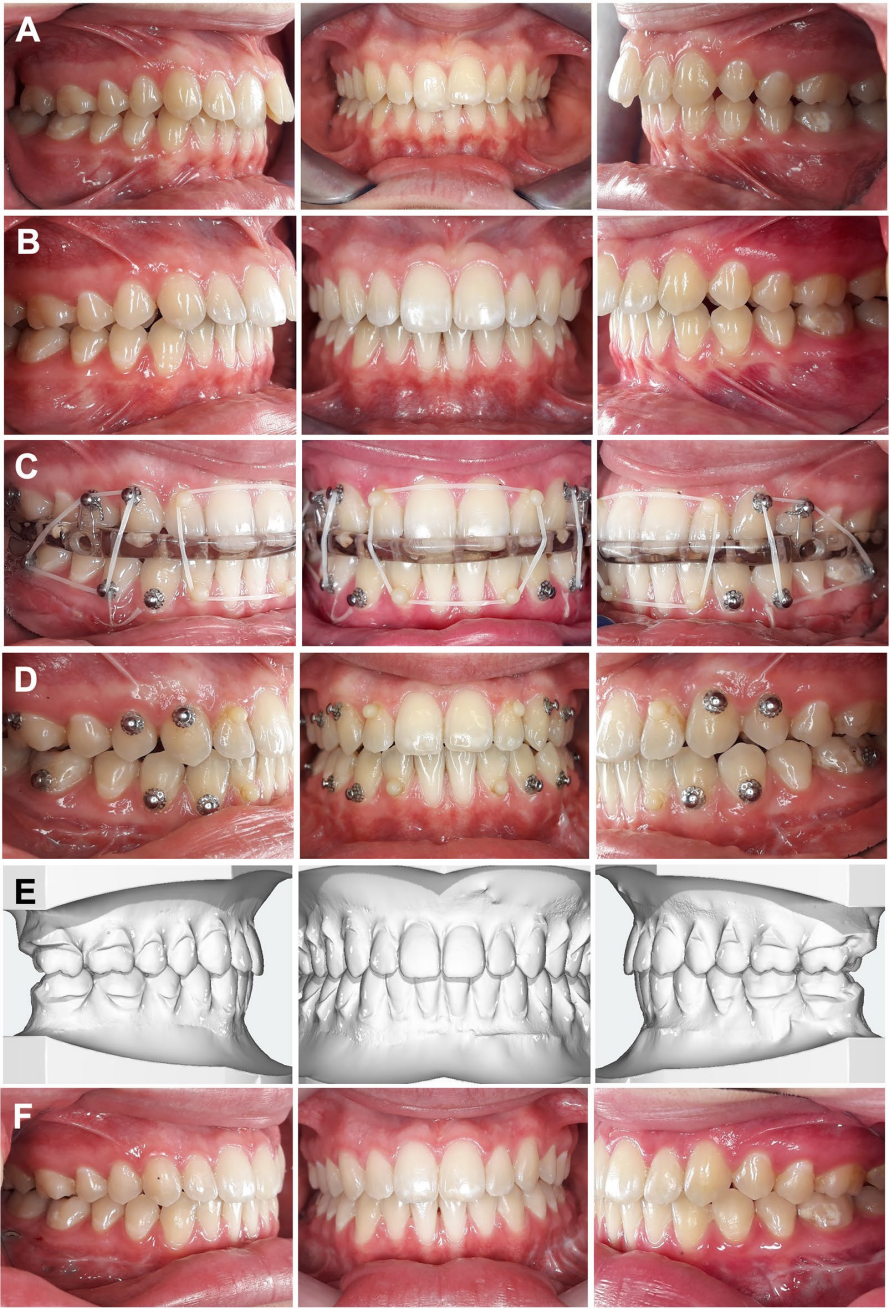
**A**/**B**: Treatment course of a 20-year-old female patient with severe Class II malocclusion managed using completely customised lingual appliances (CCLAs) as part of a combined orthodontic and surgical approach: **A**) before treatment, **B**) after decompensation, **C**) immediately after surgery with splint and intermaxillary fixation, **D**) two weeks post-surgery without splint, **E**) virtual treatment setup and **F**) after treatment and appliance removal

Twenty-five patients (12 females and 13 males) who underwent orthodontic–orthognathic treatment for manifested skeletal Class II or III malocclusion were retrospectively investigated and treated with CCLAs (WIN, DW Lingual Systems, Bad Essen, Germany) (Fig. 1). The orthodontic treatments took place at an orthodontic specialist practice (Bad Essen, Germany) or the Department of Orthodontics at Witten/Herdecke University.

The treatment was completed, and the CCLAs removed, between 2015 and 2023. The mean age at the start of treatment was 27.8 ± 10.7 years (range: 16.6–49.3 years). The mean age in the class II group was about 35.0 ± 12.8 years, and in the class III group, it was about 24.1 ± 7.1 years. The average treatment time was 2.5 ± 0.6 years, which included 1.5 ± 0.6 years for pre-surgical alignment.

**Fig. 2:**
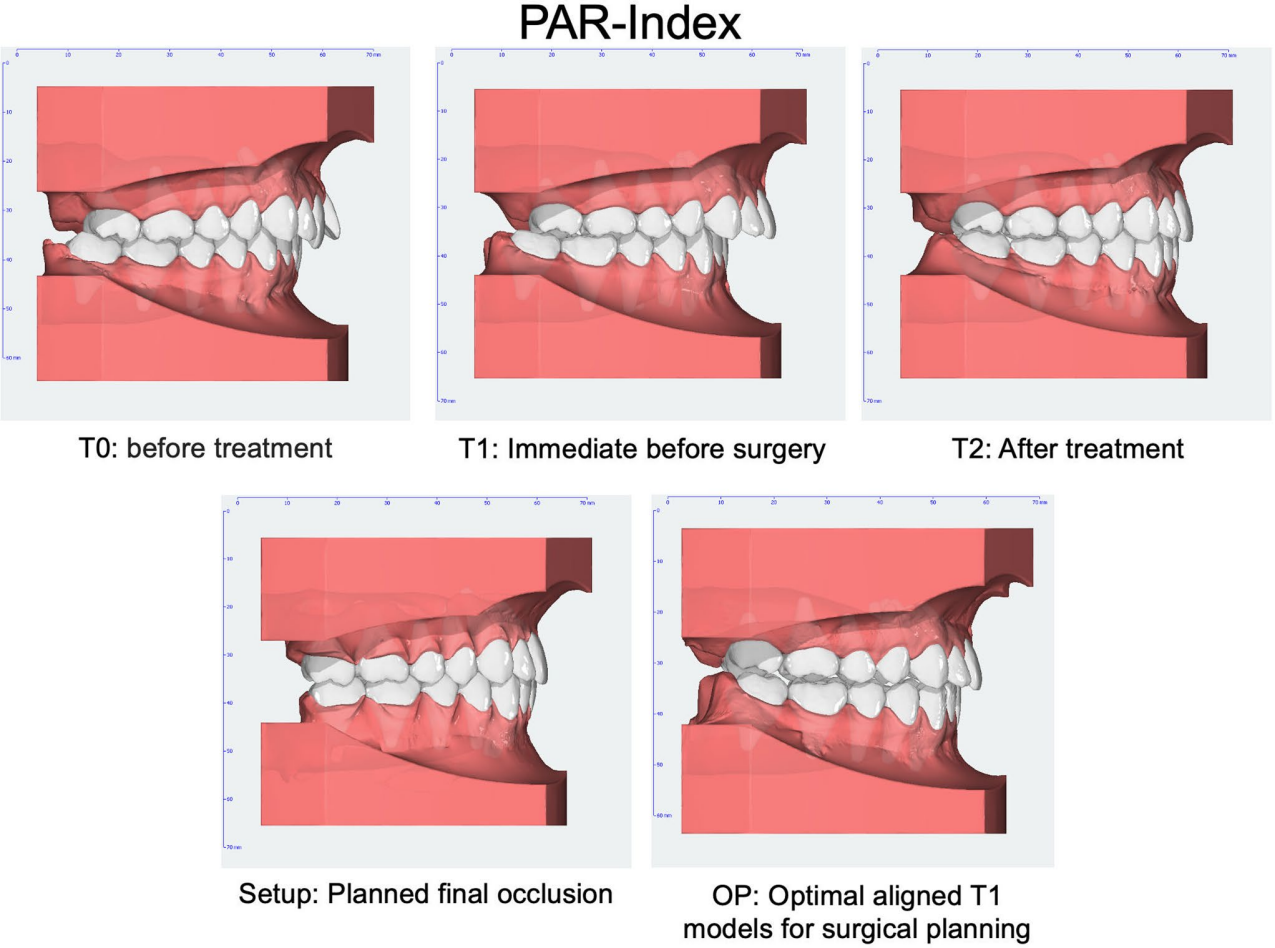
Overview of software-supported occlusal analysis using the Peer Assessment Rating (PAR) at five different stages: T0 = before treatment, T1 = immediately before surgery, T2 = after treatment, Setup = planned final occlusion, OP = operation planning

**Fig. 3:**
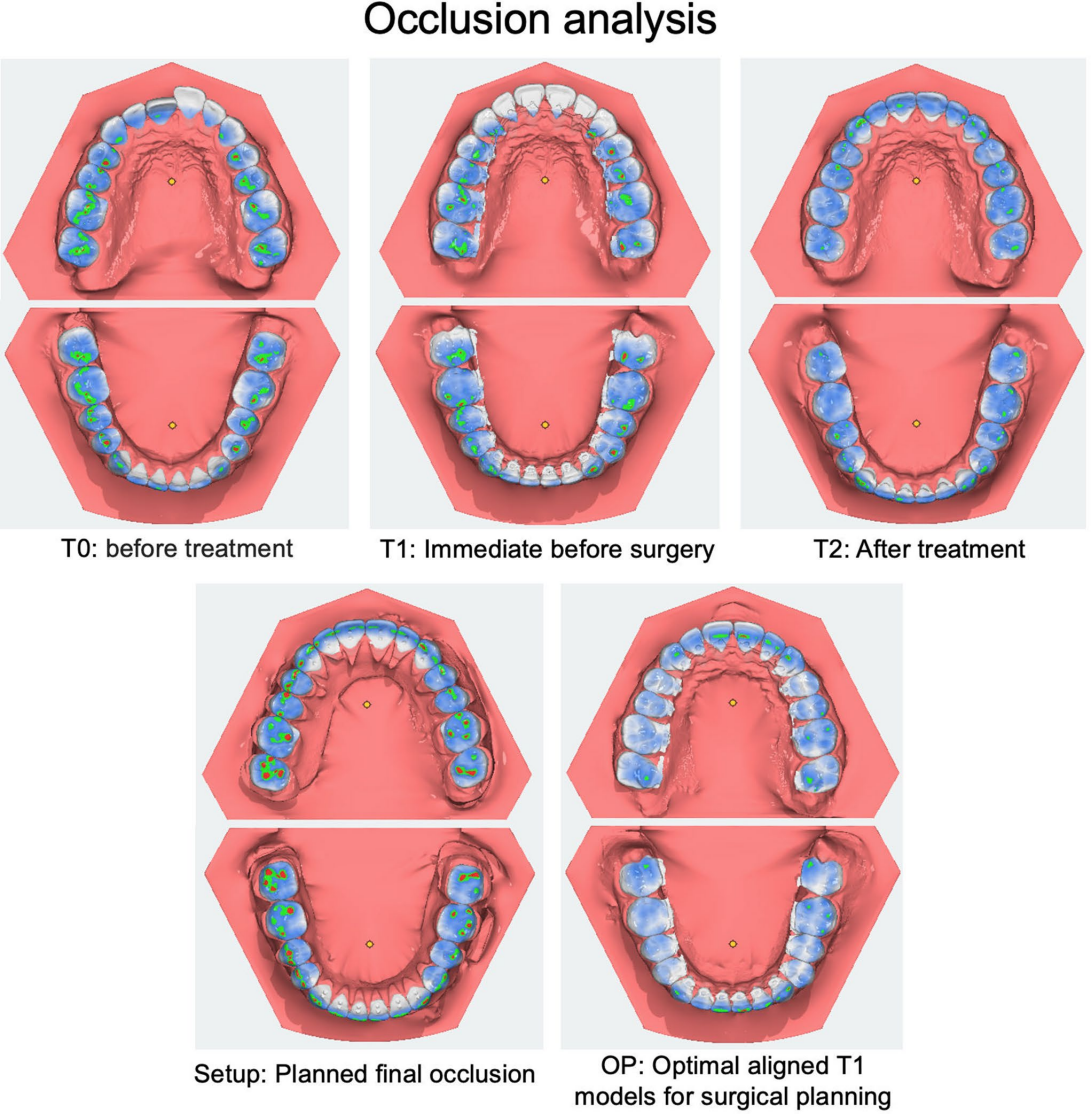
Overview of software-supported occlusal analysis by tooth pairs in contact measurements at five different stages: T0 = before treatment, T1 = immediately before surgery, T2 = after treatment, Setup = planned final occlusion, OP = operation planning

For this study, traditional plaster models of situation before treatment (T0), immediately before surgical intervention (T1) and after treatment (T2) were used. Additionally, the traditional analog setup model (Setup), which serves as the foundation for producing fully customized lingual appliances [32], and the operation model (OP), representing the conventional approach of manually planning occlusion by adjusting plaster T1 models [33], were studied (Figs. 2 and 3). These models were positioned in maximal intercuspation and subsequently digitised using an intraoral scanner (iTero Element 2, Align Technology, California, United States) after manual occlusal adjustment to ensure an accurate reproduction of occlusal relationships (Figs. 2 and 3). For both, the PAR scoring as well as the occlusal contact point analysis, the setup model serves as the reference, as it represents the best possible final occlusion.

Occlusal characteristics were then measured using the Peer Assessment Rating (PAR) Index, according to Richmond [34, 35], to generate objective data on outcome quality. Measurements were performed and verified by two calibrated investigators according to the guidelines. The following parameters were evaluated: contact point displacement in the upper and lower anterior segments, left and right buccal occlusion, overjet and anterior crossbite, overbite and frontal open bite, and centerline.

The occlusal contact conditions between the upper and lower jaws were assessed using a software program (OnyxCeph 3 Lab, Image Instruments GmbH, Chemnitz, Germany). The teeth in the virtual study models were segmented, and occlusal contacts were graphically visualised using the V.T.O. 3D Lab module. All tooth pairs in contact were then counted. Only one contact was recorded per tooth pair, even if there were multiple contact points on a single tooth.

In the patients with fully dentulous dental arches, 14 tooth contacts were possible. However, due to eight patients missing teeth, the mean maximum number of possible tooth contacts was 13.33 ± 1.11. To better assess the distribution of the tooth pairs in contact, a distinction was made between the anterior, premolar and molar segments. The maximum number of possible tooth pairs in contact was 6.00 ± 0.00 in the anterior segment (no missing front teeth), 3.64 ± 0.70 in the premolar segment and 3.68 ± 0.75 in the molar segment.

### Statistical analysis

The Shapiro–Wilk test was applied to confirm the normal distribution of the data. Due to the absence of normal distribution and the small sample sizes, statistical comparisons were performed using the paired nonparametric Wilcoxon matched-pairs signed-rank test to assess differences between the models (T0, T1, T2, Setup and OP) for each group. Additionally, the paired nonparametric Mann–Whitney test was used to compare ranks between the two groups (Class II and Class III). A power analysis is not needed due to the non-parametric tests. All analyses were conducted using Prism (version 10, GraphPad Software Inc., La Jolla, CA, USA). The level of significance was set at *p* ≤ 0.05. All results are expressed as mean ± standard deviation (SD).

### Power analysis

Post hoc power analyses were conducted using G*Power software for the Wilcoxon matched-pairs signed-rank test. An alpha level of 0.05 was applied, with sample sizes of 10 participants in Group 1 (Class II) and 15 participants in Group 2 (Class III). For the primary study aim, which evaluated the overall outcome (T0 vs. T2), the analyses determined a power of 1.00 with effect sizes of 4.6 for Class II and 3.4 for Class III. The outcome measurements were: Class II (33.3 ± 7.85 vs. 1.60 ± 2.80) and Class III (39.50 ± 11.20 vs. 0.80 ± 2.08).

For the secondary study aim, which assessed the alignment outcome (Setup vs. OP), the analyses determined a power of 0.94 with an effect size of 1.28 for Class II and a power of 0.99 with an effect size of 1.29 for Class III. The alignment outcome measurements were: Class II (0.00 ± 0.00 vs. 4.20 ± 3.29) and Class III (0.07 ± 0.26 vs. 2.47 ± 1.92).

## Results

Regarding the underlying skeletal malocclusion recorded in each case, therapeutic intervention led to statistically significant improvements in both the ANB and WITS appraisal in Class II and III patients (Table 1). When the two groups were compared, it was found that the vertical dimension was more affected in the patients with Class II malocclusion with respect to ML-NSL and ArGoGn measurements. In addition, after the treatment, both the upper and lower anterior teeth were within the physiological inclination in both groups (Table 1). Especially, a significant improvement in achieving physiological anterior tooth inclination was observed for the lower incisors (LO1-ML) in Class III patients (T0 vs. T2: 85.95 ± 7.75 vs. T2: 90.81 ± 4.08, *P* = 0.0118).

**Table 1:** Cephalometry findings and statistical comparison results for the skeletal malocclusions (Class II vs. Class III) and stages of treatment (pre-treatment [T0] vs. post-treatment [T2])

Skelettal configuration	Normal range	Class II			Class III			Class II vs. Class III	
								*p*-value	
		T0	T2	p-value	T0	T2	p-value	T0	T2
SNA	82 ± 3°	81.73 ± 4.52	81.59 ± 4.35	0.898	79.68 ± 2.90	82.51 ± 1.94	0.001*	0.200	0.967
SNB	80 ± 3°	74.97 ± 5.17	76.92 ± 4.52	0.065	83.29 ± 2.81	80.88 ± 1.59	0.0004*	< 0.0001*	0.008*
ANB	2.0 ± 2°	6.78 ± 2.12	4.73 ± 2.30	0.027	-3.61 ± 2.76	1.64 ± 1.86	< 0.0001*	< 0.0001*	0.002*
WITS	0 ± 1 mm	6.29 ± 3.56	-0.13 ± 0.94	0.002*	-10.56 ± 4.78	-3.01 ± 2.18	< 0.0001*	< 0.0001*	< 0.0001*
NL-NSL	32 ± 2°	5.46 ± 4.09	8.41 ± 4.00	0.002*	7.36 ± 2.66	8.21 ± 2.44	0.183	0.111*	0.881
ML-NSL	8.5 ± 2°	34.20 ± 11.0	37.33 ± 8.37	0.041*	31.55 ± 4.45	34.1 ± 4.26	0.002*	0.595	0.495
ML-NL	23 ± 3°	28.72 ± 10.5	28.94 ± 7.22	0.770	24.19 ± 4.64	26.0 ± 4.09	0.309	0.279	0.291
ArGoGn	128 ± 7°	121.00 ± 10.8	126.80 ± 9.57	0.006*	123.70 ± 4.79	125.80 ± 6.64	0.081	0.521	0.767
Anterior tooth angulation									
UP1-NL	112.5 ± 2°	111.90 ± 13.1	110.70 ± 4.12	0.375	116.30 ± 5.19	116.10 ± 4.40	0.8422	0.605	0.008*
LO1-ML	90 ± 3°	95.93 ± 7.81	92.09 ± 1.70	0.287	85.95 ± 7.75	90.81 ± 4.08	0.0118*	0.018*	0.374
UP1- LO1	131 ± 6°	123.40 ± 19.6	128.20 ± 6.73	0.160	132.80 ± 9.86	127.10 ± 6.79	0.0497*	0.030*	0.713

**Fig. 4:**
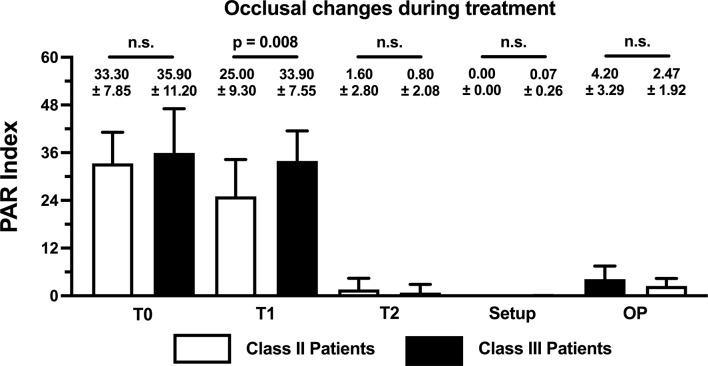
Bar chart of PAR scores with mean value and standard deviation at different stages of treatment. The p-values associated with the statistical comparisons of the skeletal malocclusion classes (Class II vs. Class III) are shown

When the dental changes were assessed using the PAR Index, a comparable great need for treatment was identified in both groups (Class II: 33.30 ± 7.85; Class III: 35.90 ± 11.20; *p* = 0.5389) (Fig. 4). However, statistically significant differences between the groups (Class II vs. Class III) were only identified during treatment at T1 when the different models were compared.

**Fig. 5:**
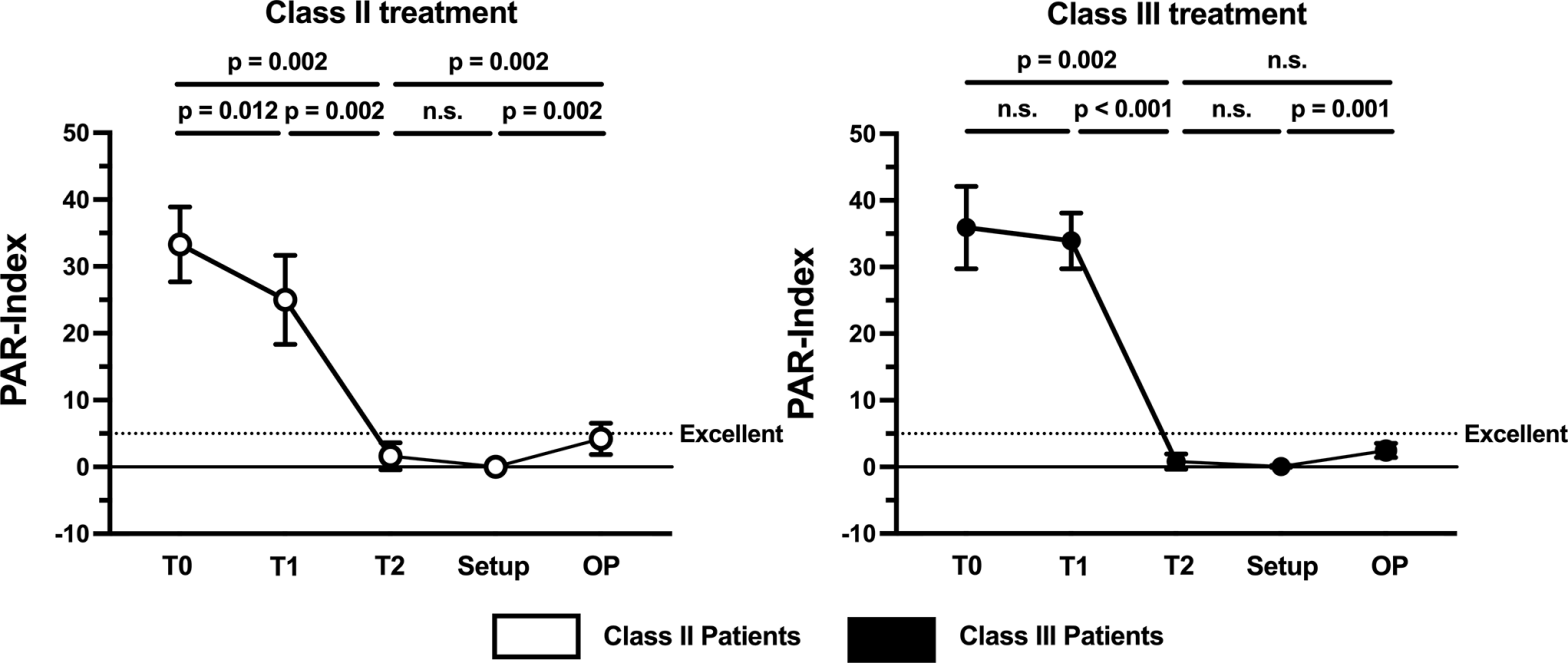
Line chart of PAR scores with mean value and 95%CI at different stages of treatment for each malocclusion. The p-values associated with the statistical comparisons of the different stages of treatment (T0, T1, T2, Setup and OP) are shown. A PAR score ≤ 5 indicates excellent occlusion

Pre-surgical orthodontic treatment led to a significant reduction in the PAR score of the patients with Class II malocclusion (T0 vs. T1: *p* = 0.012), while no significant change was observed in the patients with Class III malocclusion (T0 vs. T1: *p* = 0.534) (Fig. 5). For both malocclusions, significant changes were evident by the end of therapy (T0 vs. T2: Class II, *p* = 0.002; Class III, *p* = 0.002). However, in both groups, while no significant difference was found between the final occlusion and initial setup models (T2 vs. Setup: Class II: *p* = 0.063; Class III: *p* = 0.125), the difference between the operation planning and initial setup models was statistically significant (OP vs. Setup: Class II: *p* = 0.002; Class III: *p* = 0.001). However, all the recorded PAR scores were within the ‘excellent’ range (Fig. 5).

**Fig. 6:**
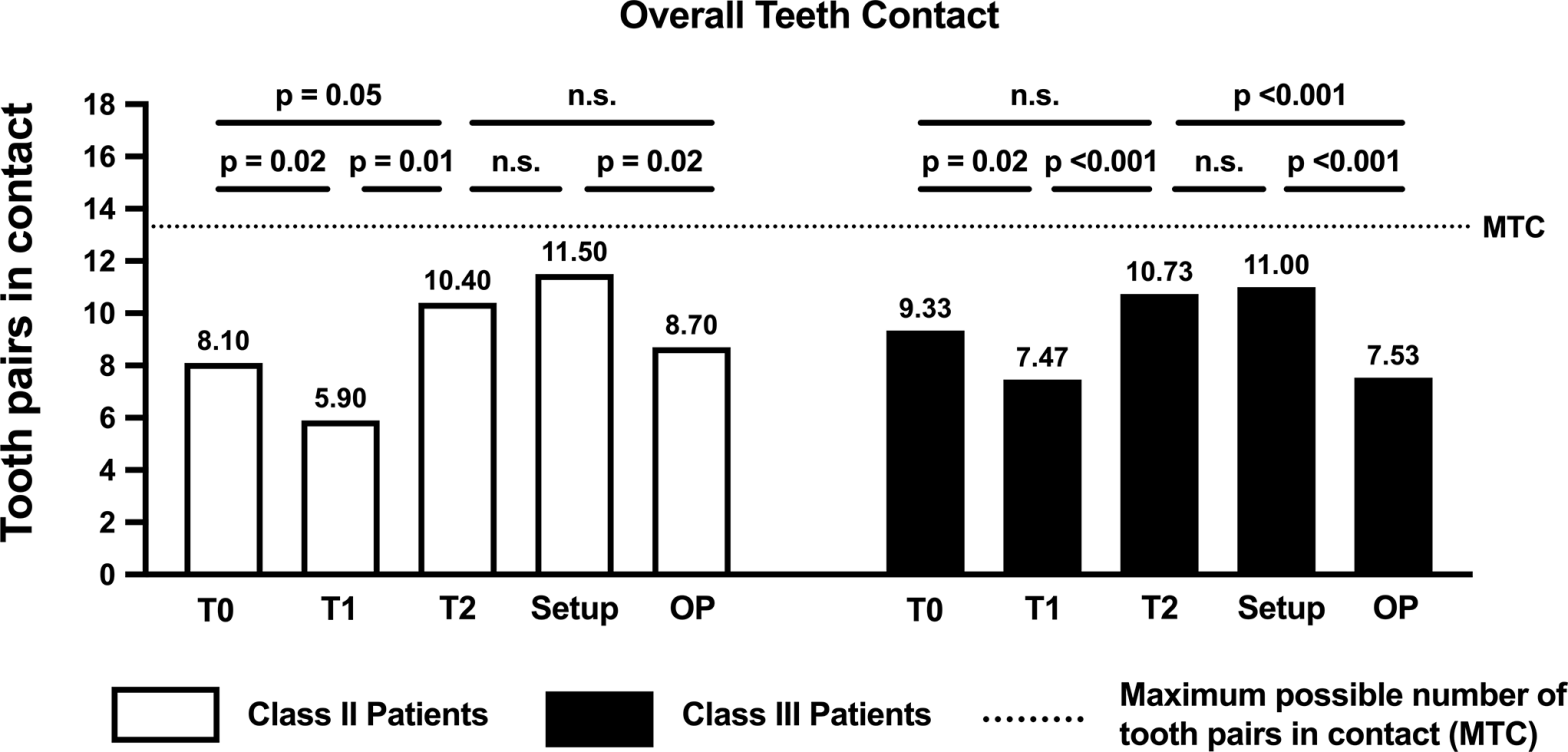
Bar chart showing the number of tooth pairs in contact at different stages of treatment for each malocclusion. The p-values associated with the statistical comparisons of the different stages of treatment (T0, T1, T2, Setup and OP) are shown. The average number of maximum tooth contacts possible (MTC) was 13.32

When the total number of tooth pairs in contact was compared between the groups (Class II vs. III), no statistically significant differences were found at any time point (Table 2). However, within each group, the occlusal tooth pairs in contact exhibited permanent and statistically significant changes (Fig. 6). Initially, the number of teeth in contact decreased during treatment (T0 vs. T1: Class II: *p* = 0.002; Class III: *p* = 0.002); however, maximum values were recorded at the end of treatment (T1 vs. T2: Class II: *p* = 0.01; Class III: *p* < 0.001). The maximum values statistically corresponded to those in the setup models in both groups (T2 vs. Setup: Class II: *p* = 0.469; Class III: *p* = 0.682). However, there were significant differences between the operation planning and setup models (OP vs. Setup: Class II: *p* = 0.016; Class III: *p* = 0.002).

**Table 2:** Comparative analysis of occlusal tooth contacts expected and achieved using CCLAs during orthognathic treatment in patients with skeletal class II and class III malocclusions (data for different tooth groups and all teeth are shown)

	Class	Anteriors		Premolars		Molars		Overall teeth	
		Teeth Contact	*P*-Value	Teeth Contact	*P*-Value	Teeth Contact	*P*-Value	Teeth Contact	*P*-Value
T0	II	1.10 ± 1.73	0.021*	3.30 ± 1.06	0.787	3.70 ± 0.68	0.218	8.10 ± 1.79	0.477
	III	3.00 ± 2.20		3.20 ± 1.01		3.27 ± 0.88		9.33 ± 3.29	
T1	II	0.10 ± 0.32	< 0.0001*	2.80 ± 1.23	0.238	3.00 ± 1.25	0.195	5.90 ± 2.13	0.082
	III	2.60 ± 1.35		2.27 ± 1.16		2.60 ± 0.63		7.47 ± 1.51	
T2	II	3.60 ± 1.84	0.170	3.40 ± 0.84	0.414	3.40 ± 0.97	0.901	10.40 ± 2.46	0.708
	III	4.60 ± 1.45		2.87 ± 1.36		3.27 ± 1.16		10.70 ± 2.60	
Setup	II	4.40 ± 1.51	0.880	3.50 ± 0.71	0.585	3.60 ± 0.97	0.629	11.50 ± 2.17	0.728
	III	4.47 ± 1.64		3.07 ± 1.22		3.53 ± 0.74		11.0 ± 2.42	
OP	II	3.50 ± 1.27	0.655	2.60 ± 1.51	0.051	2.70 ± 1.16	0.258	8.70 ± 2.45	0.297
	III	3.73 ± 1.10		1.53 ± 1.30		2.27 ± 1.10		7.53 ± 2.47	

T0: Before treatment; T1: Immediate before surgery; T2: After treatment; Setup: Optimal realigned teeth in the setup model; OP: Best possible occlusion alignment before surgical treatment; * statistically significant

**Fig. 7:**
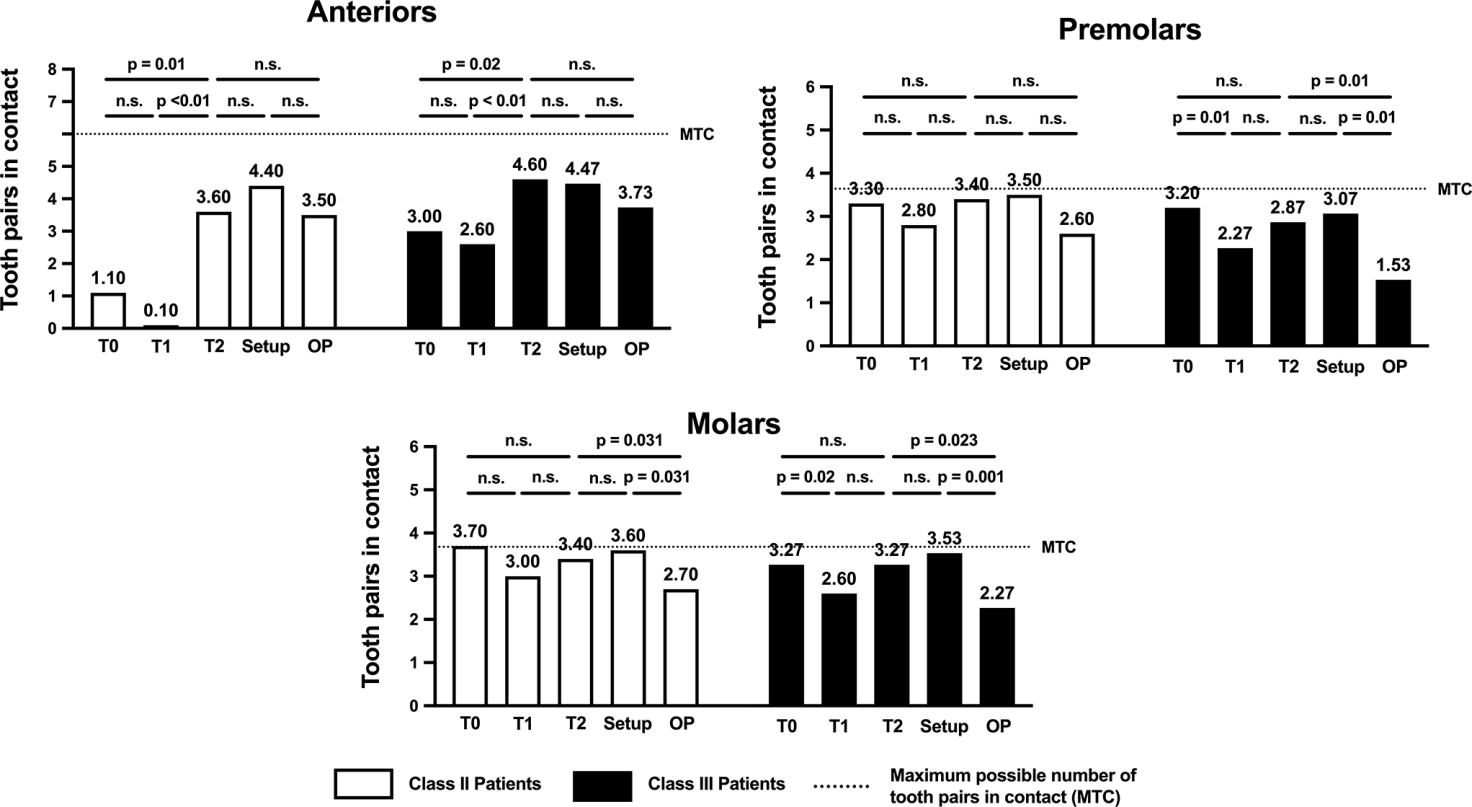
Bar chart showing the number of tooth pairs in contact in the anterior, premolar and molar segments at different stages of treatment for each malocclusion. The p-values associated with the statistical comparisons of the different stages of treatment (T0, T1, T2, Setup and OP) are shown. The MTC for the anterior, premolar and molar segments was 13.32, 3.6 and 3.7, respectively

A detailed analysis of the individual segments showed that there was an increase in the number of tooth pair contacts, particularly in the anterior segment (T0 vs. T2: Class II: *p* = 0.01, Class III: *p* = 0.02) (Fig. 7). In contrast, the number of tooth contacts remained constant in the premolar segment (T0 vs. T2: Class II: *p* > 0.999; Class III: *p* = 0.371) and the molar segment (T0 vs. T2: Class II: *p* = 0.375; Class III: *p* = 0.938). Furthermore, the number of tooth contacts in the setup models statistically corresponded to those in the post-treatment models in the premolar segment (Setup vs. T2: Class II: *p* > 0.999; Class III: *p* = 0.617) and molar segment (Setup vs. T2: Class II: *p* = 0.625; Class III: *p* = 0.500); however, the number of tooth contacts recorded in the setup models was higher than that in the operation planning models for the premolar segment (Setup vs. OP: Class II: *p* = 0.125; Class III: *p* = 0.001) and molar segment (Setup vs. OP: Class II: *p* = 0.031; Class III: *p* = 0.001). There were no statistically significant differences between the groups, except in the anterior segment measurements (Table 2).

## Discussion

Adequate pre-surgical dental arch decompensation is required to achieve an optimal outcome after surgical jaw repositioning [5, 14, 15, 36]. Unfortunately, the decompensation achieved does often not reach the required level, and insufficient orthodontic dental alignment prior to surgery can result in unfavourable baseline conditions [4]. In this context, inadequate presurgical orthodontic decompensation of the incisors has been particularly noted in the surgical treatment of Class III patients, which can limit the effectiveness of the surgical correction [37, 38]. For instance, Martinez et al. reported that in 52% of surgical cases, the upper and lower incisor decompensation did not reach ideal values, hindering the achievement of complete skeletal correction [38]. In their study that focused on incisor decompensation, Quast et al. found that incisor decompensation was insufficient in all three dimensions [5]. Although they observed no difference in the sagittal plane between patients with Class II and Class III malocclusions, a physiological overjet was more often achieved than a neutral jaw relation. In addition, Seker et al. reported insufficient incisor decompensation in the sagittal dimension and attributed this to a lack of premolar extractions [36]. In the vertical dimension, Quast et al. found that decompensation led to an increased overbite, which affected the surgical correction of the maxillomandibular plane angle in patients with a severe open bite, while in the transverse dimension, they found that midline irregularities were not adapted to the skeletal asymmetry, which resulted in improper correction of the menton [5].

Effective and precise control of the incisors, as well as a high degree of agreement between the pre-therapeutic planning and the final occlusion, can be achieved using CCLAs [7–9, 14–16]. Alouini et al. examined the controllability of incisor torque and found that the use of CCLAs resulted in a satisfactory upper incisor palatal root torque in 93.1% of cases [8]. This finding was confirmed by Fontinha et al., who reported that efficient control of the mesio-distal angulation of all anterior teeth could be achieved; however, they detected clinically significant differences (> 3°) in the torque between the setup and post-treatment tooth positions for the upper second premolars and molars [7]. Likewise, Pauls et al. reported achieving excellent incisor torque control and highly accurate predictions of the final results from the setup models [14]. In their study, the angle discrepancies of the incisors were < 3° and the translation values were < 0.3 mm, and in the lateral segments the angle discrepancies were between 3.7° and 5.18° and the translation values were between 0.26 and 0.64 mm. Thus, their findings closely aligned with those of Grauer and Proffit, who reported small discrepancies in position and rotation (generally < 1 mm and 4°, respectively), except for the second molars [16]. In this context, reference should also be made to the latest developments and trends in digital workflows for the production of 3D-printed customised lingual appliances [39], which could potentially further enhance this already high predictability.

Although the use of CCLAs is common in orthodontic treatment regimens, their use in treatment protocols that combine orthodontic and surgical components has only been reported in a limited number of case reports. There are also isolated reports of their use in association with surgery-first protocols [28, 29, 40]. These reports illustrate that combining lingual orthodontics with orthognathic surgery requires meticulous planning, including conducting a diagnostic setup and profile analysis, in close collaboration with the surgeon [26, 27, 29]. Most authors reported that this appliance was used due to patients desiring the most aesthetic treatment possible and thus inconspicuous appliances [23, 24, 26]. However, it is concerning that the current understanding of the use of CCLAs in the context of orthognathic surgery from an orthodontic standpoint is quite limited, with the majority of available literature consisting of case reports.

Therefore, in this retrospective study, we examined the quality of the occlusal changes that occurred during treatments that combined orthodontic and surgical components. Templeton et al. previously demonstrated that the PAR Index is a suitable measure for assessing outcomes and improvements in patients treated with a combined orthodontic and orthognathic approach [41]. Furthermore, to examine the occlusal changes, especially at the time of surgical bite-position correction, an occlusal analysis was carried out by observing the tooth pairs in contact. In this context, Hannebauer et al. demonstrated that PAR scoring using a fully automated method on digital study models produces very similar results compared to traditional analysis on plaster or 3D-printed study models [42]. However, while digital analysis of occlusal contacts can yield acceptable results compared to traditional analysis using articulating film [43], thin articulating paper still detects more overall contacts than digital devices, particularly in the posterior regions [44]. Nevertheless, a digital occlusion analysis was performed in this study, as we focused exclusively on tooth pairs in contact. In this case, a less precision was acceptable.

The PAR scores recorded during the different stages of treatment showed that there was statistically significant improvement before surgery (T0 vs. T1) in the patients with Class II malocclusion only. However, the 24.92% reduction in the PAR score of the patients with Class II malocclusion and the 5.57% reduction in the PAR score of the patients with Class III malocclusion before surgery indicated that no clinical improvement was achieved in either group at that time. In contrast, the 95.20% and 97.77% reductions recorded after the completion of the entire treatment regimen in the patients with Class II and Class III malocclusions, respectively, demonstrate that significant improvement can be achieved through combined orthodontic–orthognathic therapy with CCLAs in patients with severe skeletal malocclusion. This is also illustrated by the lack of statistically significant differences between the post-treatment and setup models for both types of skeletal malocclusion. Furthermore, these findings indicate that the final results of orthodontic–orthognathic therapy with CCLAs can be predicted with high accuracy from setup models. It is also worth noting that better treatment outcomes (measured using the PAR Index) have been reported in cases where CCLAs have been used in combination with orthognathic treatment compared to other appliances. For instance, 74–83.7% improvements have been achieved with conventional appliances [45, 46], whereas Kwon et al. reported an improvement of around 87% when using clear aligners [47].

The PAR scores derived from the operation planning models are particularly interesting. A score of 4.2 was recorded for the patients with Class II malocclusion, indicating an improvement of 87.39%, and a score of 2.47 was recorded for the patients with Class III malocclusion, suggesting an improvement of 93.12%. These results seem to indicate that the optimal alignment of the dental arch was achieved during the decompensation phase, even though the scores statistically significantly differed from those derived from the setup models in both groups. Nevertheless, from a clinical perspective, the scores were within the ‘excellent’ range of PAR scores (< 5) [48].

With regard to occlusal stability, because some patients were missing some teeth, the average number of possible tooth contacts was 13.32 instead of 14 (six, 3.6 and 3.7 in the anterior, premolar and molar segments, respectively). During the decompensation period (T0 vs. T1), a statistically significant reduction occurred in the number of tooth pairs in contact in both groups. By the end of the treatment, the number had significantly increased (T1 vs. T2) and was comparable to that in the setup model (T2 vs. Setup). As found in the PAR score results, in both groups, there was significantly less occlusion in the operation planning models compared to the setup models. However, at the time of surgery, in both groups, more than half of all possible tooth pairs were in contact. The detailed analysis of these contacts showed that those between anterior teeth were particularly affected by the treatment, leading to a statistically significant increase in both groups (T0 vs. T2). In contrast, the number of contacts in the premolar and molar segments remained constant. When the pre-surgical alignment was examined, it was found that the maximum number of tooth pairs in contact was achieved in the anterior segment and that fewer contact points were present in the premolar and molar segments (OP vs. Setup). This indicated that there was less occlusal contact in the posterior region before surgery than is generally expected. However, when compared to the recommendations in the literature, our results appear to be favourable [49–52]. While it has been suggested that achieving stable occlusion during surgery is crucial for postoperative stability, the definition of ‘stable occlusion’ differs, ranging from at least three-point contact [51, 52] to stable posterior occlusion [49, 50]. In this context, Liao et al. reported that stable occlusion can be achieved by five to six teeth showing occlusal contact or by occlusal contact in one, two or three segments [53]. However, it must be considered that in some treatment plans, achieving the best possible occlusion immediately after surgery is not desired. This can be the case in deep bite treatments or when surgical overcorrection is intended to prevent a relapse [2, 54]. Such adjustments are deliberately integrated into treatment planning to optimize long-term stability and functional outcomes.

With regard to the potential conclusions concerning the present results, some limitations should be mentioned. These include the fact that only patients who were treated with a CCLA were included, without comparing them to those treated with other orthodontic appliances using a preoperative setup, which may limit the generalizability of the study’s findings. However, establishing a control group would necessitate individualized setups for conventional labial appliances, which is not standard practice. Moreover, no data currently exist on fully customized vestibular appliances in the context of orthognathic treatment, making direct comparisons impossible at the moment. Likewise, the retrospective design, which introduces issues such as lack of randomization, potential selection bias, and less control over confounding variables, limits the findings.

However, despite the lack of direct comparisons, the present findings suggest that CCLAs enable highly predictable occlusal outcomes. Further studies should be conducted to compare the quality of the treatment outcomes achieved by CCLAs with other orthodontic appliances and to analyse the effects of different appliances on the surgical outcome.

## Conclusions

Considering the limitations of this investigation, including its retrospective study design, small sample size, and lack of a control group, the results indicate that using CCLAs in combined orthodontic and surgical treatment regimens for Class II and Class III patients can lead to excellent treatment outcomes, as measured by the PAR Index, and highly accurate predictions of post-treatment occlusion. Additionally, adopting this appliance can result in excellent dental arch alignment even before surgery.

## Data Availability

No datasets were generated or analysed during the current study.
